# Microbial Interactions in Oral Biofilm: Evaluating Therapeutic Interventions and the Emergence of Resistance: A Narrative Review

**DOI:** 10.7759/cureus.48021

**Published:** 2023-10-31

**Authors:** Sahil Choudhari, Jogikalmat Krithikadatta, Ipsitha Vejendla, Swathi S, Mukesh Doble

**Affiliations:** 1 Conservative Dentistry and Endodontics, Saveetha Dental College and Hospitals, Saveetha Institute of Medical and Technical Sciences (SIMATS) Saveetha University, Chennai, IND; 2 Cariology, Saveetha Dental College and Hospitals, Saveetha Institute of Medical and Technical Sciences (SIMATS) Saveetha University, Chennai, IND

**Keywords:** farnesol, resistance, multispecies, dental caries, biofilm

## Abstract

The oral cavity comprises numerous anatomical surfaces that are inhabited by a diverse array of bacteria, collectively forming a bacterial biofilm. Within this complex microbial community, certain bacterial species are etiologically linked to the development of common oral pathologies, such as dental caries and periodontitis, which stand as prominent instances of bacterial infections frequently encountered in clinical settings. Most biofilms are believed to be multispecies consortia. While single-species biofilms have been well-researched, mixed-species biofilms and their interactions amongst themselves have not drawn interest. The aim of the current review was to assess the various interactions of dual-species microorganisms in oral biofilm formation. Farnesol given exogenously for the treatment of biofilm can enhance or inhibit the growth of certain organisms, as seen in *Candida albicans*. In the age of antibiotic resistance, it is imperative to develop and uncover drugs capable of simultaneously targeting multiple species in order to mitigate antimicrobial resistance.

## Introduction and background

Microorganisms are ubiquitous in nature and play an important role in both micro- and macro-environments. Several microbes reside in the human body, particularly in mucosal areas [1], and they interact with other species in competitive situations and devise unique survival strategies in order to compete for space, nutrition, and ecological niches. Hundreds of different microbial species can be found in the oral cavity, either as planktonic cells or as biofilms, with the majority of oral microbial species being commensals [2]. Biofilms are highly organized, well-developed microbial colonies embedded inside an extracellular matrix that are responsible for a variety of human infections [3]. A biofilm may contain live and dead cells, polysaccharides, proteins, etc. Oral biofilms include aerobic and facultative anaerobic microorganisms that dwell in close proximity to one another, resulting in a variety of potential interactions that can be beneficial or harmful. Pathogenic microorganisms can cause oral infections and, in some cases, can lead to systemic disorders [2,3].

Dental biofilms represent intricate assemblies of microbial colonies interwoven with a diverse array of organic and inorganic constituents. These constituents originate from sources such as saliva, gingival crevicular fluid, and bacterial secretions, and they collectively reside within a meticulously organized polysaccharide matrix. Each microbial colony is an independent community, including different combinations of species-species interactions for adhesion and survival in the matrix. The key pathogens, including *Aggregatibacter actinomycetemcomitans*, *Porphyromonas gingivalis*, *Prevotella intermedia*, *Tannerella forsythia*, *Fusobacterium nucleatum*, Streptococcus spp., *Peptostreptococcus micros*, and *Campylobacter rectus*, along with various organic and inorganic constituents, favor the development and maintenance of the biofilm [4]. The exopolysaccharides (EPS), produced by the bacteria in the biofilm, maintain the integrity of the biofilm by preventing its desiccation and binding the essential nutrients to the matrix, enhancing the growth of microorganisms.

Streptococcus mutans is a facultatively anaerobic Gram-positive coccus with a relatively short doubling time of 1.1 hours [5]. *A. actinomycetemcomitans*, on the other hand, is a fastidious, non-spore-forming, nonmotile, facultatively anaerobic Gram-negative coccobacillus. *Porphyromonas gingivalis*, another notable microorganism, is a facultatively anaerobic Gram-negative bacterium that plays a pivotal role in the pathogenesis of periodontal disease [5]. *Candida albicans*, an opportunistic fungus, exhibits a Gram-positive nature and can exist in both unicellular (yeast) and multicellular (hyphae, pseudohyphae) forms [6]. Within the intricate oral microbiome, *Streptococcus mutans* assumes a crucial role as a prominent matrix producer. It can rapidly influence the formation of cariogenic biofilms, especially in the presence of dietary sucrose and starch, despite not necessarily being the most abundant species [7]. *Streptococcus mutans* stands as the principal causative microbial agent responsible for human dental caries. The ability of this pathogen to build and maintain a polysaccharide-encased biofilm is critical not only for its survival and persistence in the oral cavity but also for its pathogenicity. *Streptococcus mutans*-released glucosyltransferases (Gtfs) are pellicle ingredients that synthesize glucans in situ, allowing *Streptococcus mutans* and other species to colonize the area. Gtfs bind to the surfaces of other oral bacteria, turning them into glucan makers [8]. The complex and diverse society of the oral microbiome enhances the prevalence of competition among the microbial species within the biofilm. Microbial species partake in intra-specific competition within their ecological niche, contending for vital nutrients, binding sites, and their own sustenance by employing distinct competitive mechanisms. The mechanisms discussed in this context involve various processes, including the production of bacteriocins, quorum sensing (QS), the excretion of hydrogen peroxide, and the synthesis of competence-stimulating peptides (CSP). These mechanisms are utilized to gain advantages in the competition for resources and dominance. The relative vicinity of cells within and across microcolonies, which are the fundamental structural units of biofilm, creates an environment conducive to nutritional gradients, oxygen deprivation, gene transfer, and quorum sensing [9].

In the treatment of several diseases caused by pathogenic bacteria, antibiotic resistance is a vital challenge [10]. Antibiotic resistance in bacteria is complex. In bacteria, antibiotic resistance is caused by both natural defenses and genetic mutations. The presence of a wide range of resistance genes within the human microflora has been observed, indicating its potential as a reservoir for antibiotic-resistant bacteria [11]. The present review provides an overview of the interaction of microorganisms in oral biofilm formation and a comprehensive review of various assays used for assessing the impacts of dual-species microorganism interactions, which are classified based on molecular biology assays, biochemistry assays, and biofilm assays.

**Figure 1:**
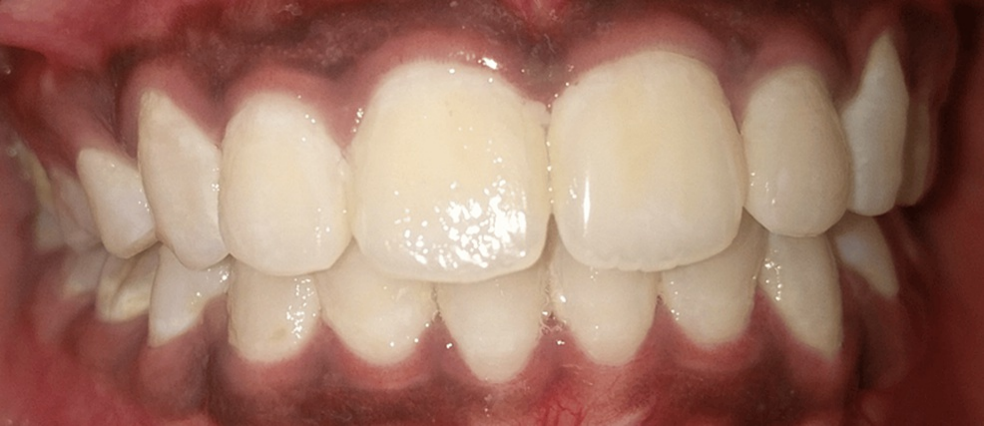
Intraoral frontal image of a patient showing plaque accumulation

**Figure 2:**
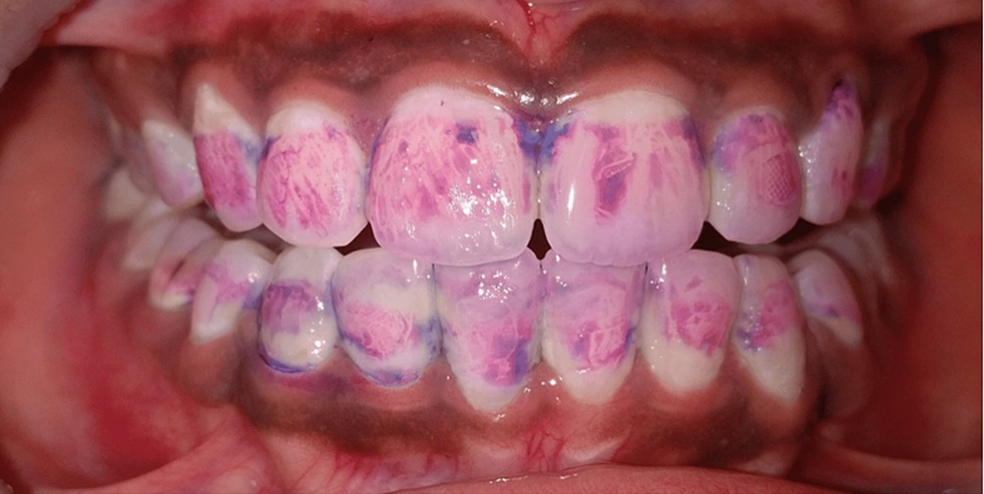
Two tone dye (Dpi Alphaplac) showing reddish pink stains depicting early biofilm which is less than 3 days old and blue stains depicting mature biofilm which is more than 3 days old

**Figure 3:**
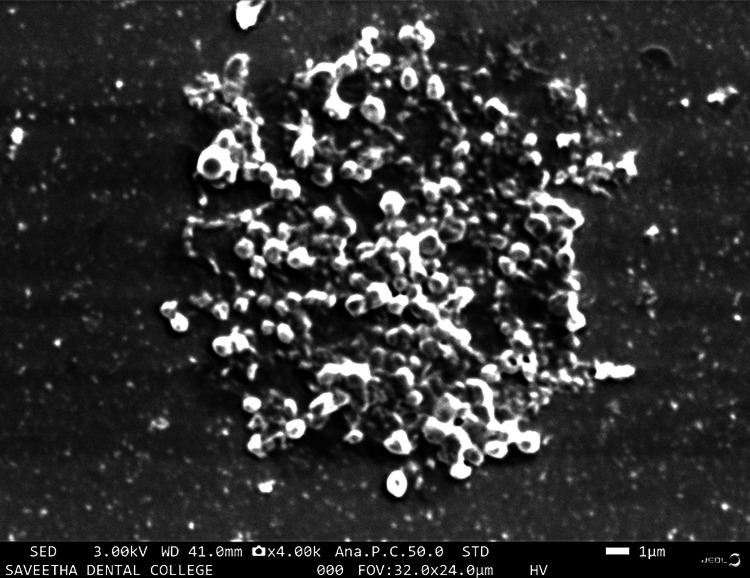
Scanning electron microscopy image of biofilm taken from a dental plaque (Mag: 4000×)

Figure 1 shows the intraoral frontal image of a patient with plaque accumulation. When a two-tone dye was applied and rinsed, the stained plaque showed reddish pink stains depicting early biofilm, which was less than three days old, and blue stains depicting mature biofilm, which was more than three days old (as shown in Figure 2). Figure 3 shows a scanning electron microscope image of a plaque sample taken from a patient, depicting bacteria with different morphologies present in it. Each species within a biofilm may inhibit or facilitate the other species. Hence, this review deals with studies involving multiple species and interactions of microorganisms present in oral biofilm and the effect of therapeutic interventions that can lead to antibiotic-resistant species.

## Review

Various microbial interactions

**Figure 4:**
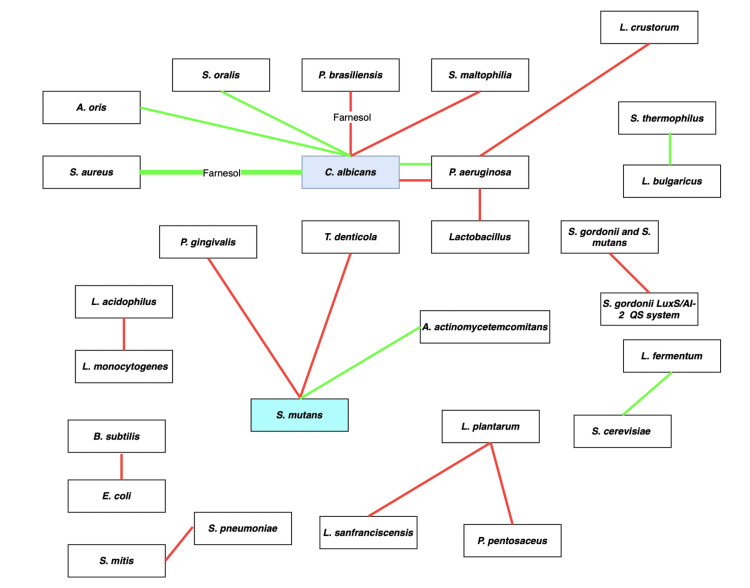
Bacterial interactions within a biofilm The red line indicates inhibition and green line indicates facilitation

Figure 4 shows a pictorial representation of the interactions of various microorganisms in a biofilm as reported in the literature. The majority of research on multispecies biofilms within the head and neck region consistently reveals the predominance of *Streptococcus mutans* and *C. albicans* as prominent members of these complex microbial communities. The following section discusses the interactions of various other organisms, focusing on the above-mentioned organisms (as shown in Figure 4). Different assays were used to assess the impacts of dual-species microorganism interactions, which were divided into three groups based on biofilm assays, molecular biology assays, and biochemistry assays. Biofilm tests involved eight studies, molecular biology assays involved nine studies, and biochemistry assays involved eight studies.

Mixed broth and agar well assays were employed to evaluate how *P. gingivalis* and *Treponema denticola* influence the properties of biofilms dependent on CSP in the context of quorum sensing within dual-species bacterial systems. The assessment of biofilm characteristics involved techniques such as crystal violet staining, DAPI staining, fluorescence microscopy analysis, and field emission scanning electron microscopy [12].

Various molecular biology assays involved in quorum sensing are differential in-gel electrophoresis for detection of protein expression, transmission electron microscopy to confirm a lack of adverse effects of farnesol on cell structure, gene expression (qRT-PCR), confocal laser scanning microscopy (architecture of the biofilms), scanning electron microscopy of the biofilm, gene expression analysis by qRNA, real-time PCR, and spectrometry followed by the plate counting method for the microbial concentration of the inoculum [13]. Transcriptomic and proteome studies, localization tests, and gel retardation assays represent a subset of the molecular assays utilized to investigate inter-microbial interactions. Other assays include the microbial viability assay, specifically the BacLight LIVE/DEAD cell viability assay, as well as the detection of anti-QS activity using the agar well diffusion method and the alpha-amylase assay [13]. In addition, the ultrastructural morphology of *P. brasiliensis* yeast cells treated with farnesol was evaluated through transmission and scanning electron microscopy [14], and the YycG autophosphorylation activities of *Staphylococcus aureus* were activated by ammonium filter binding [15]. According to biochemical assays, interspecies interactions are mediated by bacteria's well-regulated chemical production.

In a study by Peters et al., the bacterial species *Staphylococcus aureus* and the fungus species *C. albicans*, both of which are commonly found in polymicrobial biofilm communities, exhibited a biofilm architecture that involved the pathogenicity of *Staphylococcus aureus* and the hyphal components of *C. albicans* [16]. According to the findings of this study, the physical interactions and the distinctive regulation of particular virulence factors, including adhesins, immune evasion factors, and toxins, which occur as a result of polymicrobial growth, along with the inherent antibiotic resistance capabilities, may also contribute to the pathogenicity of *Staphylococcus aureus*. During coinfection, *C. albicans* and *Staphylococcus aureus*, two of the most common nosocomial infections that cause considerable morbidity, have been demonstrated to have synergistic effects, and innovative strategies may be needed in order to minimize the fungal-bacterial quorum sensing cross talk. The findings of Vila et al. [17] indicated that the chemical farnesol released by *C. albicans* suppresses the synthesis of a pigment, staphyloxanthin (STXN), that is considered to be a key virulence factor and modifies the expression of virulence-related genes in *Staphylococcus aureus*. Candidal spp. interact with *Staphylococcus epidermidis* to form biofilms. Quorum-quenching proteins QQ-5 and QQ-7 exhibit the capacity to hinder the formation of biofilms by *C. albicans* and *Staphylococcus epidermidis*. They achieve this by disrupting the yeast-to-hyphae transition in *C. albicans* and by triggering the expression of the icaR gene, responsible for encoding the repressor of polysaccharide intercellular adhesin (PIA) synthesis in *Staphylococcus epidermidis* [18]. In a study by Szafraski et al., transcriptome sequencing revealed that in the presence of *A. actinomycetemcomitans*, *Streptococcus mutans* up-regulated its entire QS regulon, suggesting that *A. actinomycetemcomitans* played an essential role in the growth of *Streptococcus mutans* [12].

*Lactobacillus crustorum* ZHG-21 demonstrated its ability to disrupt QS-regulated pathogenicity, limit biofilm production, and scavenge biofilms that have already developed [19]. According to a research study by de Rossi et al., the nosocomial pathogen *Stenotrophomonas maltophilia* interacts with *C. albicans*' pathogenicity factors, i.e., yeast-to-hyphal transition and biofilm formation, thereby down-regulating the antifungal activity [20]. In mixed biofilms, *C. albicans*-produced QS molecule farnesol may impact the pathogenicity of *Staphylococcus​​​​​​​ aureus* by acquiring a drug-tolerant phenotype, according to Kong et al. [21]. Periodontal infections inhibited *Streptococcus mutans*' quorum-sensing abilities. This might make *Streptococcus mutans* less virulent and resistant to antibacterial agents in the environment. According to Wang et al., the LuxS/AI-2 quorum-sensing system of *Streptococcus gordonii* plays a pivotal role in regulating the formation of dual-species biofilms in conjunction with *Streptococcus mutans* [22]. In another study, *L. plantarum* CY 1-1 CE exhibited significant acyl homoserine lactone (AHL) degradation activity, effectively suppressing the production of violacein, extracellular proteases, and siderophores in *Chromobacterium violaceum* CV026. The presence of *L. plantarum* CY 1-1 CE at sub-MICs (sub-minimum inhibitory concentrations) led to the suppression of biofilm production and disruption of preformed biofilms.

Commensalism has been observed between *Saccharomyces cerevisiae* and *Bacillus amyloliquefaciens* 04BBA15, where one benefits while the other is unaffected, as well as mutualism between *Saccharomyces cerevisiae* and *L. fermentum* 04BBA19, where both species mutually benefit [13]. Interestingly, in mixed cultures, there was a significant increase in α-amylase production compared to monoculture conditions. Additionally, mature Shp0316 can compensate for the absence of competence in strains of *Streptococcus thermophilus* and *Streptococcus salivarius* that are typically difficult to transform or non-transformable. In the context of phosphorylation, it was found that phosphorylated intermediate YycG in *Streptococcus pneumoniae* was not detectable in the presence of its cognate YycF, whereas both phosphorylated forms of YycG and YycF were concurrently identified in Staphylococcus aureus [15].

Yang et al. [23] found that B. subtilis MA139 inhibited E. coli K88 strongly under shaking circumstances but rather weakly under static ones. Under static circumstances, Lactobacillus alone and in conjunction with *B. subtilis* MA139 spores inhibited *Escherichia*​​​​​​​* coli* K88 effectively. Lactobacillus in conjunction with *B. subilis* spores inhibited the growth of *B. subilis* spores at a considerably higher level than Lactobacillus alone. When compared to Lactobacillus alone, *B. subtilis* MA139 considerably reduced the pH and oxidation-reduction potential of the co-culture broth. Falkler and Burger [24] discovered that *Streptococcus mutans* cultured at a higher sucrose concentration absorbed less *F. nucleatum*.

Influence of farnesol

Farnesol (3,7,11-trimethyl-2,6,10-dodecatriene-1-ol) is an extracellular quorum-sensing molecule (QSM) generated constantly in biofilms throughout growth at temperatures ranging from 23 to 43 °C and serving as an anti-biofilm agent by suppressing filamentation in *C. albicans*, which is essential for biofilm formation [25]. Farnesol reduced *Streptococcus mutans* biofilm acid generation but had no effect on *C. albicans* hydrolytic enzyme production at subinhibitory concentrations. It effectively disrupts and prevents adhesion in *F. keratoplasticum* biofilms. Farnesol has the ability to disrupt the biofilm by breaking the extracellular matrix. In the presence of exogenously supplemented farnesol or farnesol secreted by *C. albicans* in biofilm, *Staphylococcus aureus* has exhibited significantly enhanced tolerance to antimicrobials. [26]. Farnesol enhances and inhibits the growth of certain organisms, as shown in Figure 4. Farnesol is more effective at destroying cell membranes, as assessed by live/dead staining, than at killing biofilm bacteria, which may cause biofilm separation and hence reduce biofilm biomass. Moreover, it also plays a role in biofilm detachment.

Various assays and clinical tests used in detecting biofilms

There are several physical, chemical, morphological, and biological assays used for detecting biofilms on surfaces, which are listed in Table 1.

**Table 1:** Various assays and tests with their clinical applications

Assays and tests	Clinical application
Gel Retardation Assay	To investigate the formation of protein DNA complexes in a crude nuclear protein extract
Baclight Live/Dead Cell Viability Assay	To assess the viability of bacterial populations as a function of the membrane integrity of the cell
Alpha-Amylase Assay	Determination of α-amylase activity
Agra-Gfp Reporter Assay	Visualize spatial and temporal patterns of gene expression in vivo
Co-Culture Assay	To study the interactions between cell populations
Mixed Broth Assay	Determine the lowest concentration of the assayed antimicrobial agent
Agar Well Assay	used to evaluate the antimicrobial activity of plants or microbial extracts
Crystal Violet Staining	To stain the nuclei of adherent cells. Works as an intercalating dye and allows the quantification of DNA which is proportional to the number of cells.
Dapi Staining	To determine the number of nuclei and to assess gross cell morphology.
Fluorescence Microscopic Analysis	To view features of small specimens such as microbes. Also used to visually enhance 3D features at small scales.
Scanning Electron Microscopy	To view surface's topography and composition
Differential In-Gel Electrophoresis	To assess changes in protein abundance
Transmission Electron Microscopy	View thin specimens through which electrons can pass generating a projection image
qRT-PCR	Relative and absolute quantification of gene expression. Validation of DNA micro-array results.
Confocal Laser Scanning Microscopy	Optical slicing through tissue
Spectrometry	Detect, determine, or quantify the molecular and/or structural composition of a sample

Resistance

The biofilm mode of organization provides microbes with various survival advantages, the most significant of which is the development and spread of antibiotic resistance. Bacteria within biofilms exhibit significantly greater antibiotic resistance than their freely suspended, planktonic counterparts [27]. Targeting the virulence properties of potential pathogens is a popular alternative to antimicrobials in the battle against antimicrobial resistance nowadays.

Although the precise molecular mechanisms responsible for antibiotic resistance remain elusive, various pathways have been implicated. These pathways include mechanisms that provide protection against oxidative stress, the expression of efflux pumps, the protective barrier created by extracellular polymeric substance (EPS) components, heterogeneous subpopulation growth patterns, as well as processes involving the volatilization, precipitation, chelation, and chemical modification of antimicrobial drugs to hinder their diffusion reactions [28]. *Pseudomonas aeruginosa* is developing resistance to all known conventional antimicrobial drugs, posing a serious hazard to human health. Antibiotic resistance is a serious concern in *P. aeruginosa* infections, especially in immunocompromised individuals, where the infectious organisms can readily take over the host's cellular machinery while sheltering in QS-driven biofilms [29].

Future scope

Comprehending the mechanism behind biofilm formation in multispecies organisms holds the potential to pave the way for innovative approaches to biofilm management. Such insights can be invaluable in devising novel strategies to control and combat bacterial biofilm growth in clinical settings. Understanding how the biofilm phenotype differs from the planktonic phenotype will aid in developing new strategies against multispecies biofilm. Farnesol formed in biofilm inhibits and functions as an antibiofilm activity in *C. albicans*, *Streptococcus mutans*, *Staphylococcus epidermidis*, and *Fusarium keratoplasticum* biofilm growth [25], as well as having the ability to rupture the biofilm by breaking the extracellular matrix. The need for culture-independent molecular-based techniques, like metagenomics, is imperative to obtain a comprehensive evaluation of the presence of a wide range of suspected pathogens in samples. Due to the intricate nature of these biofilms, interpreting the results can be challenging. Therefore, future research should prioritize exploring emerging approaches capable of identifying biological activity and functions within oral biofilms. Most existing diagnostic tools primarily target individual organisms. However, it is likely that future diagnostic procedures will necessitate a fundamental shift in our understanding of diseases. This shift will focus on recognizing microbial communities and their roles within oral biofilms, moving beyond traditional microbiological classifications to an understanding of their functions. Formulating drugs that target a mixed biofilm rather than a single species will aid in better disease control, prevent the emergence of antibiotic-resistant strains, and improve the prognosis.

## Conclusions

In conclusion, the current review focuses on multi-species oral biofilms, emphasizing the need for improved assays to identify and study diverse organisms within these biofilms. The current review also highlights the importance of developing drugs capable of targeting multiple species simultaneously to combat antimicrobial resistance. Computational approaches are proposed to unravel resistance mechanisms within biofilms. While this review mainly covers aerobic and facultative anaerobic organisms, there is a clear call for innovative techniques to investigate anaerobic biofilm contributors. This review provides valuable insights for advancing our comprehension of oral biofilms and improving therapeutic strategies by tackling these challenges.

## References

[1] Wu L, Luo Y (2021). Bacterial quorum-sensing systems and their role in intestinal bacteria-host crosstalk. Front Microbiol.

[2] Berger D, Rakhamimova A, Pollack A, Loewy Z (2018). Oral biofilms: development, control, and analysis. High Throughput.

[3] Bowen WH, Burne RA, Wu H, Koo H (2018). Oral biofilms: pathogens, matrix, and polymicrobial interactions in microenvironments. Trends Microbiol.

[4] Saini R, Saini S, Sharma S (2011). Biofilm: a dental microbial infection. J Nat Sci Biol Med.

[5] Beckers HJ, van der Hoeven JS (1982). Growth rates of Actinomyces viscosus and Streptococcus mutans during early colonization of tooth surfaces in gnotobiotic rats. Infect Immun.

[6] Bhardwaj RG, Ellepolla A, Drobiova H, Karched M (2020). Biofilm growth and IL-8 &amp; TNF-α-inducing properties of Candida albicans in the presence of oral gram-positive and gram-negative bacteria. BMC Microbiol.

[7] Paes Leme AF, Koo H, Bellato CM, Bedi G, Cury JA (2006). The role of sucrose in cariogenic dental biofilm formation--new insight. J Dent Res.

[8] Bowen WH, Koo H (2011). Biology of Streptococcus mutans-derived glucosyltransferases: role in extracellular matrix formation of cariogenic biofilms. Caries Res.

[9] Donlan RM (2002). Biofilms: microbial life on surfaces. Emerg Infect Dis.

[10] Grosjean H (2009). DNA and RNA Modification Enzymes: Structure, Mechanism, Function and Evolution.

[11] Sommer MO, Church GM, Dantas G (2010). The human microbiome harbors a diverse reservoir of antibiotic resistance genes. Virulence.

[12] Szafrański SP, Deng ZL, Tomasch J (2017). Quorum sensing of Streptococcus mutans is activated by Aggregatibacter actinomycetemcomitans and by the periodontal microbiome. BMC Genomics.

[13] Fossi BT, Tavea F, Fontem LA, Ndjouenkeu R, Wanji S (2014). Microbial interactions for enhancement of α-amylase production by Bacillus amyloliquefaciens 04BBA15 and Lactobacillus fermentum 04BBA19. Biotechnol Rep (Amst).

[14] Derengowski LS, De-Souza-Silva C, Braz SV, Mello-De-Sousa TM, Báo SN, Kyaw CM, Silva-Pereira I (2009). Antimicrobial effect of farnesol, a Candida albicans quorum sensing molecule, on Paracoccidioides brasiliensis growth and morphogenesis. Ann Clin Microbiol Antimicrob.

[15] Clausen VA, Bae W, Throup J, Burnham MK, Rosenberg M, Wallis NG (2003). Biochemical characterization of the first essential two-component signal transduction system from Staphylococcus aureus and Streptococcus pneumoniae. J Mol Microbiol Biotechnol.

[16] Peters BM, Jabra-Rizk MA, Scheper MA, Leid JG, Costerton JW, Shirtliff ME (2010). Microbial interactions and differential protein expression in Staphylococcus aureus -Candida albicans dual-species biofilms. FEMS Immunol Med Microbiol.

[17] Vila T, Kong EF, Ibrahim A (2019). Candida albicans quorum-sensing molecule farnesol modulates staphyloxanthin production and activates the thiol-based oxidative-stress response in Staphylococcus aureus. Virulence.

[18] Weiland-Bräuer N, Malek I, Schmitz RA (2019). Metagenomic quorum quenching enzymes affect biofilm formation of Candida albicans and Staphylococcus epidermidis. PLoS One.

[19] Cui T, Bai F, Sun M, Lv X, Li X, Zhang D, Du H (2020). Lactobacillus crustorum ZHG 2-1 as novel quorum-quenching bacteria reducing virulence factors and biofilms formation of Pseudomonas aeruginosa. LWT.

[20] de Rossi BP, García C, Alcaraz E, Franco M (2014). Stenotrophomonas maltophilia interferes via the DSF-mediated quorum sensing system with Candida albicans filamentation and its planktonic and biofilm modes of growth. Rev Argent Microbiol.

[21] Kong EF, Tsui C, Kucharíková S, Van Dijck P, Jabra-Rizk MA (2017). Modulation of Staphylococcus aureus response to antimicrobials by the Candida albicans quorum sensing molecule farnesol. Antimicrob Agents Chemother.

[22] Wang X, Li X, Ling J (2017). Streptococcus gordonii LuxS/autoinducer-2 quorum-sensing system modulates the dual-species biofilm formation with Streptococcus mutans. J Basic Microbiol.

[23] Yang JJ, Niu CC, Guo XH (2015). Mixed culture models for predicting intestinal microbial interactions between Escherichia coli and Lactobacillus in the presence of probiotic Bacillus subtilis. Benef Microbes.

[24] Falkler WA, Burger BW (1981). Microbial surface interactions: reduction of the haemagglutination activity of the oral bacterium Fusobacterium nucleatum by absorption with streptococcus and bacteroides. Arch Oral Biol.

[25] Rodrigues CF, Černáková L (2020). Farnesol and tyrosol: Secondary metabolites with a crucial quorum-sensing role in Candida biofilm development. Genes (Basel).

[26] Kischkel B, Souza GK, Chiavelli LU, Pomini AM, Svidzinski TI, Negri M (2020). The ability of farnesol to prevent adhesion and disrupt Fusarium keratoplasticum biofilm. Appl Microbiol Biotechnol.

[27] Saxena P, Joshi Y, Rawat K, Bisht R (2019). Biofilms: architecture, resistance, quorum sensing and control mechanisms. Indian J Microbiol.

[28] Flemming HC (2016). EPS-then and now. Microorganisms.

[29] Bhardwaj S, Bhatia S, Singh S, Franco F Jr (2021). Growing emergence of drug-resistant Pseudomonas aeruginosa and attenuation of its virulence using quorum sensing inhibitors: a critical review. Iran J Basic Med Sci.

